# Trends in radiotherapy administration in the management of hepatocellular carcinoma: Analysis of a Korean tertiary hospital registry of hepatocellular carcinoma patients diagnosed between 2005 and 2017

**DOI:** 10.3389/fonc.2022.928119

**Published:** 2022-07-22

**Authors:** Bong Kyung Bae, Hee Chul Park, Jeong Il Yu, Gyu Sang Yoo, Dong Hyun Sinn, Moon Seok Choi, Joo Hyun Oh

**Affiliations:** ^1^ Department of Radiation Oncology, Samsung Medical Center, Sungkyunkwan University School of Medicine, Seoul, South Korea; ^2^ Department of Medicine, Samsung Medical Center, Sungkyunkwan University School of Medicine, Seoul, South Korea

**Keywords:** hepatocellular carcinoma, radiotherapy, trend, three-dimensional conformal radiotherapy, intensity-modulated radiotherapy, stereotactic body radiotherapy, proton beam therapy

## Abstract

**Purpose:**

To present the trends in radiotherapy for the management of hepatocellular carcinoma (HCC) at a single tertiary referral hospital in South Korea.

**Materials and Methods:**

We retrospectively reviewed prospectively collected registry data of patients newly diagnosed with HCC between January 2005 and December 2017 at the Samsung Medical Center. Trends in radiotherapy, delivery techniques, tumor stage, and age were evaluated.

**Results:**

During the study period, 9,132 patients were newly diagnosed with HCC at our institution. Of these, 2,445 patients (26.8%) received radiotherapy for all lesions, including extrahepatic metastases; 1,865 patients (20.4%) received radiotherapy for intrahepatic lesions alone, and 469 patients (5.1%) received radiotherapy as initial management. Although the proportion of patients receiving radiotherapy increased slightly over the study period (24.2% vs. 26.6%), the proportions of patients receiving radiotherapy for intrahepatic lesions (16.8% vs. 21.9%) and as initial management (0.1% vs. 12.5%) increased dramatically. The majority of patients treated between 2005 and 2008 received three-dimensional conformal radiotherapy (56.3%), whereas the majority of patients treated between 2018 and 2021 received proton beam therapy (43.6%). With the technical developments, the overall survival (OS) of patients who received radiotherapy as initial management increased significantly (5-year OS: from 5.4% to 30.1%), and the OS difference between patients who did and did not receive radiotherapy as initial management significantly decreased (ratio of restricted mean survival time: from 0.383 to 0.544).

**Conclusion:**

This registry-based, retrospective study indicated an increasing trend in the utilization of radiotherapy, adoption of advanced radiotherapy techniques, and OS improvements in patients with HCC.

## Introduction

Primary liver cancer is the sixth most commonly diagnosed cancer and the third leading cause of cancer-related death worldwide. Hepatocellular carcinoma (HCC) accounts for 75–85% of all cases of primary liver cancer (1). Most cases of HCC develop from chronic liver disease. Risk factors for chronic liver disease include hepatitis B virus (HBV) infection, hepatitis C virus (HCV) infection, alcohol consumption, and aflatoxin exposure. In Asian countries, except Japan, and Africa, the predominant risk factor for HCC is HBV infection (70%), whereas HCV infection appears to be the key risk factor in Western countries and Japan (50–70%) (2, 3).

Curative treatments for HCC include liver transplantation, resection, and ablative therapies. However, only 30–40% of patients with HCC are diagnosed in the early stages of the disease and are eligible for curative treatment (2, 3). Even with curative therapy other than liver transplantation, more than half of patients develop recurrence (4–6). For the management of patients with intermediate- or higher-stage disease or recurrent disease, other treatment options, such as radiotherapy, trans-arterial chemoembolization (TACE), chemotherapy, and immunotherapy, should be considered.

Although HCC is a radiosensitive tumor, radiotherapy for intrahepatic HCC lesions is limited owing to the radiosensitive nature of the normal liver parenchyma (7, 8). Hence, radiotherapy has historically been administered only for palliative treatment in patients with symptomatic metastatic lesions and in patients with advanced HCC with major vascular tumor thrombi (9, 10). However, with advances in radiotherapy, stereotactic body radiotherapy (SBRT) and intensity-modulated radiotherapy (IMRT) with photon or proton beam therapy (PBT) have been introduced for the management of intrahepatic HCC lesions and have favorable outcomes with minimal toxicity (11–15). Based on these advances, several guidelines for HCC recommend that radiotherapy can be considered for patients for whom other curative therapies are not suitable (16, 17), and the role of radiotherapy in the management of HCC has changed and grown.

However, there has been no overall review on how these changes affect actual clinical practice. To better understand how the changing landscape has influenced the utilization of radiotherapy, we aimed to present the observed trends in the administration of radiotherapy for the management of HCC in a single tertiary referral hospital in South Korea.

## Materials and methods

With approval of the Institutional Review Board (IRB number 2021-12-093-001), prospectively collected registry data for patients with newly diagnosed, previously untreated HCC at the Samsung Medical Center were analyzed in this study. The HCC diagnosis was confirmed either histologically or clinically, based on the guidelines of the Korean Liver Cancer Association-National Cancer Center (16, 18, 19). Clinical diagnosis of HCC was made based on the typical imaging hallmarks of HCC on multiphase CT or multiphase MRI. The major imaging feature for HCC diagnosis are defined as arterial phase hyper-enhancement with washout in portal venous, delayed or hepatobiliary phases. When imaging-based diagnosis was inconclusive or atypical features were seen, pathological diagnosis with biopsy was indicated. Data from the following patients were entered into the registry: (1) patients who were not previously treated for HCC; (2) patients who received at least one treatment or care for HCC at our institution; and (3) patients who had not been newly diagnosed or had undergone treatment for a malignancy other than HCC at the time of registration. The patients’ baseline characteristics were collected, including age, sex, Eastern Cooperative Oncology Group performance status (ECOG PS), viral etiology, Child-Pugh classification, albumin-bilirubin (ALBI) grade, Barcelona Clinic Liver Cancer (BCLC) stage, modified Union for International Cancer Control (mUICC) stage, and initial treatment method. The details of the HCC registry have been previously described (20). Data collected from patients registered between January 2005 and December 2017 were analyzed.

During the study period, as previously mentioned in other study from our institution, treatment of HCC was performed according to “Practice Guidelines for the Management of Hepatocellular Carcinoma” published by the Korean Liver Cancer Association-National Cancer Center Korea (16, 18–20). Surgical resection or radiofrequency ablation (RFA) was the primary treatment option for early-stage disease. Clinicians considered TACE and/or radiotherapy for patients with marginal liver function and HCC located in areas unsuitable for RFA. TACE was primarily considered for intermediate- or locally advanced-stage disease. Consolidative radiotherapy was added to treat hepatic lesions for patients with large tumors, macroscopic vascular/ductal invasion or an insufficient response to TACE. As atezolizumab plus bevacizumab, or lenvatinib were not available at the time of enrollment, sorafenib was prescribed for patients with metastatic or extensive tumors. When treatment of choice was evident based on the guidelines, the primary physician of the first visit decided the treatment option for the patient. If there were controversial issues in decision-making, the multidisciplinary team decided the treatment modality for the patient. The details of the multidisciplinary team approach for HCC in our institution had been described previously (21).

Registered patients were reviewed to determine whether they received radiotherapy for HCC management during follow-up. Radiotherapy was categorized into three groups based on the timing and target lesion. Radiotherapy utilization was defined as radiotherapy administered at any time during the HCC treatment period for the management of any lesion, including extrahepatic metastatic sites. Radiotherapy for intrahepatic lesions was defined as radiotherapy administered at any time during the HCC treatment period for the management of intrahepatic lesions. Radiotherapy as the initial management for intrahepatic lesions was defined as adoption of radiotherapy in the course of initial management at the time of diagnosis; this includes treatment using TACE plus consolidative radiotherapy, systemic therapy plus radiotherapy, palliative radiotherapy alone, and definitive radiotherapy alone. For patients who underwent multiple radiotherapy sessions, each session was counted individually. Radiotherapy delivery techniques were stratified into the following five subtypes: two-dimensional (2D) radiotherapy, three-dimensional conformal radiotherapy (3D-CRT), IMRT, SBRT, and PBT.

The radiotherapy utilization rate was obtained by dividing the number of patients who received radiotherapy by the total number of patients whose data were entered into the registry and was expressed as a percentage. Trends in radiotherapy utilization rates were plotted based on the year of diagnosis. Temporal trends in radiotherapy utilization were plotted based on the time from diagnosis to radiotherapy administration, with cumulative and differential counts. Trends in radiotherapy delivery technique, BCLC stage, and age at radiotherapy were plotted based on the time of radiotherapy administration.

Differences in clinical characteristics according to radiotherapy utilization were compared using the chi-square test. The Cochran–Armitage test was performed to assess the trend of radiotherapy utilization. Overall survival (OS) was calculated and compared between patients who received radiotherapy as initial treatment and those who received other treatment options for initial management. The log rank test was used to compare survival differences between groups. To observe the changing trends in the survival difference based on the initial treatment method, the 5-year estimated value of restricted mean survival time (RMST), which indicates the area under the survival curve, was analyzed (22). The RMST ratio was calculated to determine the trends of differences in OS between the groups over time. Statistical significance was set at p < 0.05. Statistical analyses were performed using SPSS Statistical software ver. 27.0 (IBM, Inc., Armonk, NY, USA) and R studio ver. 1.3.1093 (R Foundation for Statistical Computing, Vienna, Austria; http://www.r-project.org).

## Results

### Patients

During the inclusion period, data from 9,132 patients were entered into the HCC registry. Radiotherapy was administered to 2,445 patients (26.8%; 3,570 lesions) during the entire HCC treatment period. Radiotherapy for intrahepatic lesions was administered to 1,865 patients (20.4%; 2,144 lesions), of whom 469 (5.1%; 469 lesions) received radiotherapy as the initial treatment for HCC. **Supplementary Table 1** summarizes the clinical characteristics of patients based on radiotherapy status during the entire HCC treatment period. In overall HCC registry, patients with male sex (80.0%), positive for HBV (73.4%), had an ECOG PS 0 (91.6%), diagnosed clinically (94.6%), with liver function of Child–Pugh classification A (86.0%), and whose HCCs were classified as mUICC stage II (39.0%) and BCLC stage A (41.7%) were predominant. In the patients who received radiotherapy, the proportion of male sex, better underlying liver function, and worse tumor stage was higher.

**Supplementary Table 2** shows the utilized radiotherapy techniques by the BCLC stage of the disease. The proportion of early disease, BLCL stages 0 and A, was higher for highly conformal radiotherapy techniques: 39.8% of 2D; 39.3% of 3D-CRT; 54.0% of IMRT; 63.5% of SBRT; 64.7% of PBT, respectively. The proportion of worse disease status, BCLC stages C and D, was higher for conventional radiotherapy techniques: 44.5% of 2D; 47.5% of 3D-CRT; 32.6% of IMRT; 22.7% of SBRT; 18.2% of PBT, respectively.

### Trends of radiotherapy utilization

**Figure 1:**
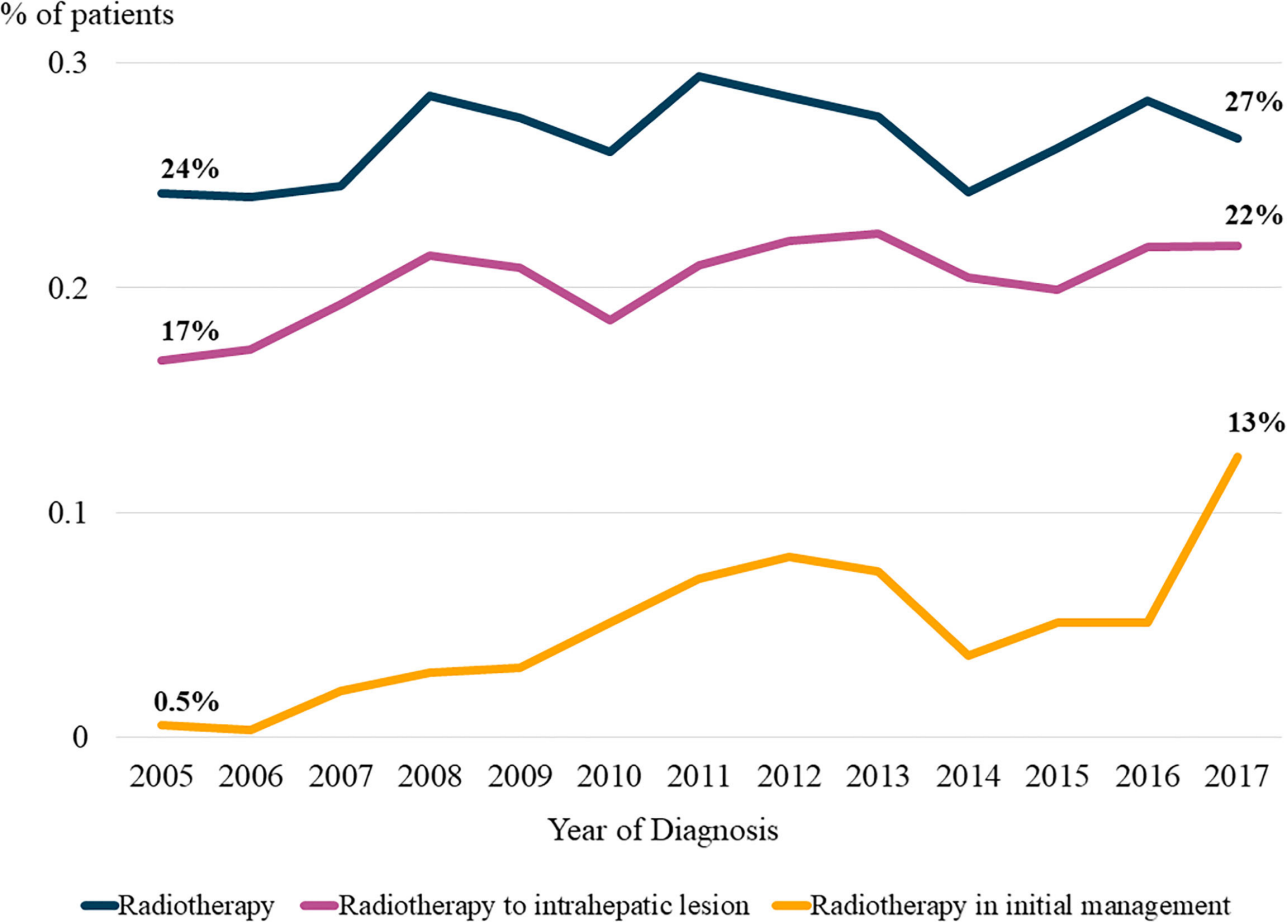
Trends in the radiotherapy utilization rate. The proportions of patients in the registry who received radiotherapy based on the year of diagnosis are plotted in line graph.

The trends in radiotherapy utilization rate over the entire HCC management period based on the year of HCC diagnosis are presented in **Figure 1**. The overall radiotherapy utilization rate was relatively consistent throughout the study period (p=0.239). Approximately 24% of patients diagnosed in 2005 received radiotherapy, whereas 27% of patients diagnosed in 2017 received radiotherapy. The rate of radiotherapy administration for intrahepatic lesions significantly increased over the study period (p=0.006). Approximately 17% of patients diagnosed in 2005 received radiotherapy for intrahepatic lesions, whereas 22% of patients diagnosed in 2017 received radiotherapy for intrahepatic lesions. Although there were only marginal increases in the rates of radiotherapy utilization for all lesions and intrahepatic lesions (3% and 5% increases, respectively), there was a dramatic increase in the utilization of radiotherapy for the initial management of HCC (p<0.001). While only 0.5% of patients diagnosed in 2005 received radiotherapy as initial treatment, 13% of patients diagnosed in 2017 received radiotherapy as initial treatment. The results of the Cochran–Armitage test are summarized in **Supplementary Table 3**.

**Figure 2:**
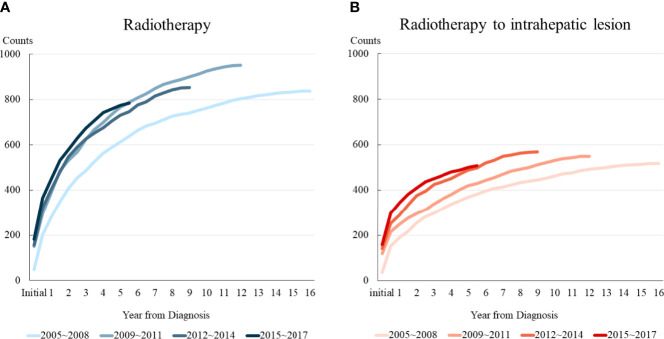
Temporal trends in radiotherapy utilization based on the time from diagnosis. Patients were divided into four groups according to the year of diagnosis. **(A)** Radiotherapy for all lesions, and **(B)** Radiotherapy for intrahepatic lesions.

**Figure 2** shows the temporal trends in radiotherapy utilization based on the interval from the date of HCC diagnosis to the date of radiotherapy for HCC management, counting each radiotherapy session individually. The slope of the graph was steeper for patients who were diagnosed more recently, indicating that the number of patients who received radiotherapy at the same time point after diagnosis gradually increased over time. Consistent trends were observed in the rates of overall radiotherapy utilization (**Figure 2A**) and radiotherapy for intrahepatic lesions (**Figure 2B**). Differential counts showed that the number of patients who received radiotherapy peaked in the first year after diagnosis and continued to decrease over time (**Supplementary Figure 1**).

**Table 1** shows the trends in the characteristics of patients who received radiotherapy for intrahepatic lesions as the initial management. Only 37 patients (7.9%) enrolled between 2005 and 2008 received radiotherapy as initial management, whereas 165 patients (35.2%) enrolled between 2015 and 2017 received radiotherapy as initial management for intrahepatic lesions. Most patients had a relatively good performance status, with BCLC stage C and Child–Pugh classification A liver function throughout the study period. Furthermore, the adoption of advanced radiotherapy delivery techniques has increased over time. Approximately 97.3% of patients enrolled between 2005 and 2008 received 3D-CRT, whereas 21.2% of patients enrolled between 2015 and 2017 received PBT.

**Table 1:** Characteristics of patients who received radiotherapy as initial management for intrahepatic lesions.

Characteristics	Year of diagnosis			
	2005–2008	2009–2011	2012–2014	2015–2017
N. of patients	37 (7.9%)	123 (26.2%)	144 (30.7%)	165 (35.2%)
Age (median, range)	53 (26–73)	54 (23–74)	55 (27–84)	57 (30–87)
ECOG PS				
0	25 (67.6%)	111 (90.2%)	140 (97.2%)	133 (80.6%)
1	11 (29.7%)	11 (9.0%)	2 (1.4%)	30 (18.2%)
2	1 (2.7%)	0 (0.0%)	1 (0.7%)	1 (0.6%)
3	0 (0.0%)	1 (0.8%)	1 (0.7%)	1 (0.6%)
4	0 (0.0%)	0 (0.0%)	0 (0.0%)	0 (0.0%)
BCLC stage				
O	0 (0.0%)	0 (0.0%)	2 (1.4%)	2 (1.2%)
A	2 (5.4%)	10 (8.2%)	5 (3.5%)	11 (6.7%)
B	1 (2.7%)	1 (0.8%)	3 (2.1%)	9 (5.5%)
C	34 (91.9%)	111 (90.2%)	132 (91.6%)	138 (83.6%)
D	0 (0.0%)	1 (0.8%)	2 (1.4%)	5 (3.0%)
ALBI grade				
I	11 (29.7%)	87 (70.7%)	101 (70.1%)	100 (60.6%)
II	25 (67.6%)	35 (28.5%)	41 (28.5%)	62 (37.6%)
III	1 (2.7%)	1 (0.8%)	2 (1.4%)	3 (1.8%)
Child-Pugh classification				
A	31 (83.8%)	114 (92.7%)	127 (88.2%)	141 (85.5%)
B	6 (16.2%)	9 (7.3%)	16 (11.1%)	20 (12.1%)
C	0 (0.0%)	0 (0.0%)	1 (0.7%)	4 (2.4%)
Radiotherapy technique				
2D	1 (2.7%)	1 (0.8%)	0 (0.0%)	0 (0.0%)
3D-CRT	36 (97.3%)	120 (97.6%)	134 (93.1%)	112 (67.9%)
IMRT	0 (0.0%)	2 (1.6%)	8 (5.5%)	13 (7.9%)
SBRT	0 (0.0%)	0 (0.0%)	2 (1.4%)	5 (3.0%)
PBT	0 (0.0%)	0 (0.0%)	0 (0.0%)	35 (21.2%)
Initial treatment				
TACE + consolidative radiotherapy	26 (70.3%)	118 (95.9%)	133 (92.3%)	130 (78.8%)
Systemic therapy + radiotherapy	2 (5.4%)	0 (0.0%)	1 (0.7%)	5 (3.0%)
Palliative radiotherapy alone	9 (24.3%)	5 (4.1%)	7 (4.9%)	15 (9.1%)
Definitive radiotherapy alone	0 (0.0%)	0 (0.0%)	3 (2.1%)	15 (9.1%)

ECOG PS, Eastern Cooperative Oncology Group performance status; BCLC, Barcelona Clinic Liver Cancer; ALBI, albumin-bilirubin; 3D-CRT, 3D-conformal radiotherapy; IMRT, intensity modulated radiotherapy; SBRT, stereotactic body radiotherapy; PBT, proton beam therapy; TACE, trans-arterial chemoembolization.

**Supplementary Figure 2** shows the trends in radiotherapy utilization rate with patients grouped based on initial treatment: initial hepatic resection or other initial treatments. While proportion of patients receiving radiotherapy constantly increased in the patients who underwent initial other treatments, proportion of patients receiving radiotherapy after initial hepatic resection was relatively consistent throughout the period.

### Trends of clinical factors

**Figure 3:**
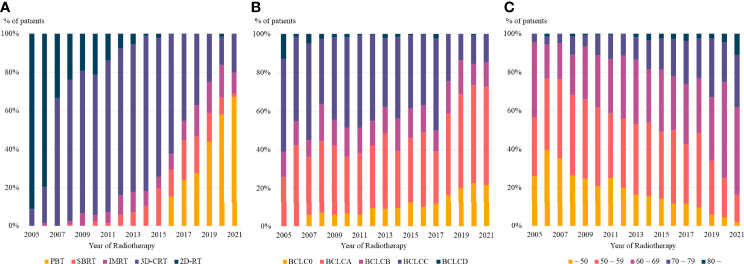
The trends in **(A)** radiotherapy delivery technique, **(B)** BCLC stage, and **(C)** age at radiotherapy based on the year of radiotherapy. PBT, proton beam therapy; SBRT, stereotactic body radiotherapy; IMRT, intensity modulated radiotherapy; 3D-CRT, three dimensional conformal radiotherapy, 2D-RT, two dimensional radiotherapy; BCLC, Barcelona Clinic Liver Cancer.

Radiotherapy delivery techniques changed dramatically during the study period. Patients who received radiotherapy in 2005 were treated with 2D- or 3D-CRT, whereas more than half of those treated in 2021 received PBT. Taken together, more than 80% of the radiotherapy delivery methods used for the management of HCC in 2021 were highly conformal radiotherapy, including IMRT, SBRT, and PBT (**Figure 3A**).

Moreover, there was a consistent change in the disease stages of patients who received radiotherapy. Patients treated in 2005 had relatively advanced disease, with no patients having BCLC stage 0, whereas more than 20% of patients treated in 2021 had BCLC stage 0 (**Figure 3B**). The age of patients who received radiotherapy also showed a consistent pattern, with a continuously increasing number of older patients treated with radiotherapy in recent years (**Figure 3C**).

### Trends of overall survival

**Figure 4:**
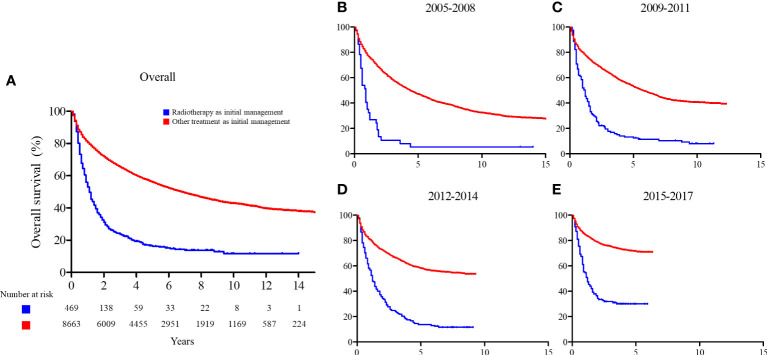
Overall survival comparison between patients who received radiotherapy as initial management and those who did not. **(A)** Entire cohort; **(B)** patients enrolled between 2005 and 2008; **(C)** patients enrolled between 2009 and 2011; **(D)** patients enrolled between 2012 and 2014; and **(E)** patients enrolled between 2015 and 2017.

The 2-year and 5-year OS rates of the patients who received radiotherapy as initial treatment and those who did not were significantly different (2-year: 31.2% vs. 71.9% and 5-year: 16.2% vs. 56.0%; p < 0.001, respectively). We found a consistent improvement in OS for patients who received radiotherapy as initial treatment over time, from 13.5% and 5.4% in 2005 to 34.3% and 30.1% in 2017 for 2-year and 5-year OS, respectively. A similar change was seen for patients who did not receive radiotherapy with a change from 67.2% and 53.1% in 2005 to 78.7% and 71.6% in 2017 for 2-year and 5-year OS, respectively. The ratio of RMST increased over time (from 0.383 to 0.544), indicating that the difference in OS between patients who received radiotherapy as initial management and those who did not decreased over time. The OS curves and RMST results are summarized in **Figure 4** and **Table 2**.

**Table 2:** Overall survival and RMST values of patients over time.

		Overall survival			RMST	
		2-year	5-year	P value	Estimated	Ratio
Overall	Initial RT (+)	31.2%	16.2%	< 0.001		
	Initial RT (-)	71.9%	56.0%			
2005-2008	Initial RT (+)	13.5%	5.4%	< 0.001	1.257	0.383
	Initial RT (-)	67.2%	47.0%		3.281	
2009-2011	Initial RT (+)	29.1%	12.5%	< 0.001	1.712	0.493
	Initial RT (-)	70.9%	53.1%		3.471	
2012-2014	Initial RT (+)	34.4%	13.9%	< 0.001	1.881	0.526
	Initial RT (-)	72.5%	58.1%		3.573	
2015-2017	Initial RT (+)	34.3%	30.1%	< 0.001	2.143	0.544
	Initial RT (-)	78.7%	71.6%		3.938	

RMST, restricted mean survival time; RT, radiotherapy.

Initial radiotherapy technique was also observed to be related with OS. The OS rates of the patients who received highly conformal radiotherapy (IMRT, SBRT, or PBT) as initial treatment were significantly better than the patients who received conventional radiotherapy (2D or 3D-CRT), irrespective of the stage of patients (5-year OS of overall population: 33.0% vs. 11.4%, p < 0.001; BCLC stage 0 or A: 85.6% vs. 20.0%, p < 0.001; BCLC stage B or higher: 21.7% vs. 11.0%, p = 0.004, respectively). The OS curves are shown in **Supplementary Figure 3**.

## Discussion

This retrospective study demonstrated changes in the role of radiotherapy in the management of HCC over the last 16 years at our institution. The use of radiotherapy has increased over time, particularly in regard to radiotherapy for intrahepatic lesions and radiotherapy as initial management. There has also been a dramatic increase in the use of highly conformal radiotherapy and PBT. Our results demonstrated the changing role of radiotherapy in the management of HCC.

In the past, radiotherapy played a minimal role in the management of HCC, especially in the management of intrahepatic lesions, owing to the occurrence of radiation-induced toxic events such as radiation-induced liver disease (23). Previously, radiotherapy was mainly used for palliation, management of patients with portal vein tumor thrombus, and symptomatic extrahepatic metastases. However, with recent advances in radiotherapy delivery and image-guidance techniques, radiotherapy for intrahepatic lesions is viewed as an effective and safe treatment modality. Several prospective studies on SBRT as treatment for HCC patients with Child–Pugh classification A or B liver function reported good oncologic outcomes (2-year local control rates ranging from 80.9% to 97%) with acceptable toxicity (rates of grade 3 or higher gastrointestinal or liver toxicity ranging from 0% to 7%) (12, 24–27). Two prospective studies on PBT also reported good results, with 3-year local control rates of 95.2% and 94.5% and no severe toxic events (28, 29). The details of these prospective studies are summarized in **Supplementary Table 4**.

Previous studies have compared radiotherapy with traditional curative treatment options. Su et al. (30) compared SBRT with liver resection for patients with small HCC and Child–Pugh classification A liver function. No difference was observed in OS or progression-free survival between the two treatment methods, indicating that the local effects of SBRT were similar to those of liver resection. Kim et al. (31) conducted a multinational study to compare the effectiveness of SBRT and RFA for unresectable HCC. Their results favored SBRT over RFA, particularly for larger tumors in the subphrenic region, and for the tumors that progressed after TACE. Kim et al. (13) compared the outcomes of PBT and RFA in patients with recurrent or residual HCC in a prospective phase 3 non-inferiority trial and reported that the local progression-free survival rate of patients undergoing PBT was non-inferior to that of patients undergoing RFA.

In addition to the aforementioned prospective studies, numerous retrospective studies have also reported the effectiveness of radiotherapy as a treatment for HCC. Based on these results, the current National Comprehensive Cancer Network guidelines recommend SBRT as an alternative to ablation/embolization techniques or when these therapies have failed or are contraindicated (32). Also, the American Society for Radiation Oncology clinical practice guideline provided evidence-based recommendation for radiotherapy in HCC recently, strongly recommending radiotherapy as a potential first-line treatment in patients with liver-confined HCC who are not candidates for curative therapy, as consolidative therapy after incomplete response to liver-directed therapies, and as a salvage option for local recurrences (33).

The baseline clinical characteristics of the patients who received radiotherapy in this study differed significantly from those of the patients who did not receive radiotherapy (**Supplementary Table 1**). The reason for the patients who received radiotherapy had relatively preserved liver function status and advanced disease could be owing to the indications for radiotherapy itself. Radiotherapy is relatively contraindicated for patients with poor liver function, and patients with early-stage disease are generally candidates for resection or ablative treatments. However, the reason for the increased proportion of male sex and HBV infection is difficult to define. Male sex and HBV infection are known to be poor prognostic factors for survival and recurrence (34, 35). The poor prognosis for these patients may have influenced the increased proportion of these patients in the radiotherapy arm as they may have required radiotherapy for further cancer management. However, it is difficult to draw concrete conclusions regarding the reason for this difference, and further analysis is required.

The proportion of patients who received radiotherapy as initial management for intrahepatic lesions dramatically increased over time in the current study, whereas the overall radiotherapy utilization rate and the rate of radiotherapy for intrahepatic lesions were relatively consistent. These proportions may not have noticeably increased because of the relatively short follow-up duration of the patients whose data were entered into the registry in more recent years. The data of patients entered the registry in 2005 were reviewed to determine whether they received radiotherapy within 16 years after diagnosis, whereas the data of patients entered the registry in 2017 were only reviewed to determine whether they received radiotherapy within 4 years after diagnosis. Some patients whose data were recently entered into the registry are likely to receive radiotherapy with further follow-up; therefore, the overall radiotherapy utilization rate and radiotherapy administration for intrahepatic radiotherapy are expected to increase in future.

High conformal radiotherapy techniques, PBT, IMRT and SBRT, are generally considered as main radiotherapy techniques for HCC. However, in our institution, there was marked increase in the proportion of PBT, but the proportion of IMRT and SBRT were relatively constant or even decreased over time (**Figure 3A**). The reason for this difference seems to be due to the treatment policy of our institution. When radiotherapy for intrahepatic lesion was required, PBT was preferred after installation of proton center. As a consequence, the proportion of SBRT and IMRT showed relatively consistent trend over time. There was ups and downs in the proportion of individual radiotherapy techniques, but it is to note that the proportion of high conformal radiotherapies increased considerably over time.

We have shown that the difference in OS between patients who did and did not receive radiotherapy as initial management for HCC decreased over time (**Figure 2** and **Table 4**). However, the management of HCC generally involves prolonged management with multiple treatments. And as other treatment modalities also advanced over past decade, consideration of the initial management alone may not reflect the clinical outcomes. However, all patients were further managed with the most appropriate treatment options based on their status, irrespective of the initial treatment method. It seems noticeable that survival differences between groups have narrowed down in this clinical setting; however, the current results should be interpreted with caution.

The current study has some limitations. First, as a single-institutional retrospective study of HBV endemic area, the treatment policy at our institution may not be identical to that at other institutions. Also, while all HCC treatment options are covered by the National Health Insurance Service in Korea, the coverage may differ in other geographical areas. Therefore, the proportion of patients receiving radiotherapy and radiotherapy delivery methods in other institutions may not be the same as those observed in the current study. However, we believe that although the numbers may not be identical, trends in the increased utilization of radiotherapy in the management of HCC will be observed in other institutions with recent prospective trials confirming the effectiveness of RT in HCC management and recommendations of the guidelines reflecting these outcomes (11, 13, 16, 17, 24, 26, 32, 36). Second, the current study did not discuss the response to radiotherapy or radiotherapy-related toxicity. As the aim of current study was to report the changing trends of radiotherapy utilization in the management of HCC, our study includes various treatment settings from palliative to definitive, with heterogeneous radiotherapy doses and techniques over more than 15 years. It was not possible to report the treatment response and toxicity due to the heterogeneity. However, as shown in **Supplementary Table 4**, in the current era of radiotherapy, radiotherapy is a safe and considerable treatment option in the management of HCC. Third, while we have addressed the changing trends of radiotherapy utilization and radiotherapy techniques, the recurrences and the managements of new recurrences were not covered in current study. Analysis of pattern of recurrences and the managements of recurrences, such as resection, liver transplantation, RFA, TACE, and chemotherapy, would have added additional depth to the study, but was not available currently. Future prospective studies might be needed to clarify these limitations.

In conclusion, the use of radiotherapy for HCC management at our institution increased dramatically from 2005 to 2017. The number and proportion of patients receiving radiotherapy for intrahepatic lesions as initial management and highly conformal radiotherapy are increasing continuously. Taken together, the findings of the current study show increased utilization of radiotherapy in the management of HCC and a shift in the paradigm of radiotherapy from palliative management of extrahepatic lesions and vascular invasions to curative treatment of intrahepatic lesions.

## Data availability statement

The original contributions presented in the study are included in the article/**supplementary material**. Further inquiries can be directed to the corresponding authors.

## Ethics statement

This study was approved by Institutional Review Board of Samsung Medical Center (SMC IRB 2021-12-093-001). The study adhered to World Medical Association's Declaration of Helsinki for Ethical Human Research. The informed consent was waived due to the retrospective nature of the study.

## Author contributions

Conceptualization, HP. Data curation, BB, HP, JY, GY, DS, MC, and JO. Formal analysis, BB, JY, and HP. Investigation, BB, JY, and HP. Methodology, BB, JY, and HP. Funding acquisition, JY. Project administration, MC, and HP. Manuscript writing—original draft, BB, and JY. Manuscript writing—review and editing, BB, HP, GY, MC, JO, and JY. All authors have read and agreed to the published version of the manuscript.

## Funding

This research was supported by the Basic Science Research Program through the National Research Foundation of Korea (NRF), which is funded by the Ministry of Education (NRF-2022R1C1C1005415).

## Conflict of interest

The authors declare that the research was conducted in the absence of any commercial or financial relationships that could be construed as a potential conflict of interest.

## Publisher’s note

All claims expressed in this article are solely those of the authors and do not necessarily represent those of their affiliated organizations, or those of the publisher, the editors and the reviewers. Any product that may be evaluated in this article, or claim that may be made by its manufacturer, is not guaranteed or endorsed by the publisher.

## Supplementary material

Supplementary Figure 1 Differential counts of radiotherapy utilization based on time from diagnosis. **(A)** radiotherapy to all lesions; **(B)** radiotherapy to intrahepatic lesions.

Supplementary Figure 2 Trends in radiotherapy utilization rate with patients grouped based on initial treatment: initial hepatic resection or other initial treatments. Trends of initial hepatic resection group is shown in solid line, and other initial treatment group is shown in dotted line.

Supplementary Figure 3 Overall survival curves of patients who received radiotherapy as initial management, categorized based on radiotherapy techniques. **(A, B)** overall patients; **(C, D)** BCLC stage 0 or A patients; **(E, F)** BCLC stage B or higher patients. 3D-CRT, 3D-conformal radiotherapy; IMRT, intensity modulated radiotherapy; SBRT, stereotactic body radiotherapy; PBT, proton beam therapy

## References

[1] Sung H Ferlay J Siegel RL Laversanne M Soerjomataram I Jemal A. Global cancer statistics 2020: GLOBOCAN estimates of incidence and mortality worldwide for 36 cancers in 185 countries. CA Cancer J Clin (2021) 71(3):209–49. doi: 10.3322/caac.21660 33538338

[2] Chen LT Martinelli E Cheng AL Pentheroudakis G Qin S Bhattacharyya GS. Pan-Asian adapted ESMO clinical practice guidelines for the management of patients with intermediate and advanced/relapsed hepatocellular carcinoma: a TOS-ESMO initiative endorsed by CSCO, ISMPO, JSMO, KSMO, MOS and SSO. Ann Oncol (2020) 31(3):334–51. doi: 10.1016/j.annonc.2019.12.001 32067677

[3] European Association For The Study Of The L European Organisation For R, Treatment Of C. EASL-EORTC clinical practice guidelines: management of hepatocellular carcinoma. J Hepatol (2012) 56(4):908–43. doi: 10.1016/j.jhep.2011.12.001 22424438

[4] Dhir M Melin AA Douaiher J Lin C Zhen WK Hussain SM. A review and update of treatment options and controversies in the management of hepatocellular carcinoma. Ann Surg (2016) 263(6):1112–25. doi: 10.1097/SLA.0000000000001556 26813914

[5] Sapisochin G Bruix J. Liver transplantation for hepatocellular carcinoma: outcomes and novel surgical approaches. Nat Rev Gastroenterol Hepatol (2017) 14(4):203–17. doi: 10.1038/nrgastro.2016.193 28053342

[6] Wang MD Li C Liang L Xing H Sun LY Quan B. Early and late recurrence of hepatitis b virus-associated hepatocellular carcinoma. Oncologist (2020) 25(10):e1541–e51. doi: 10.1634/theoncologist.2019-0944 PMC7543359 32472951

[7] Dawson LA Ten Haken RK. Partial volume tolerance of the liver to radiation. Semin Radiat Oncol (2005) 15(4):279–83. doi: 10.1016/j.semradonc.2005.04.005 16183482

[8] Pan CC Kavanagh BD Dawson LA Li XA Das SK Miften M. Radiation-associated liver injury. Int J Radiat Oncol Biol Phys (2010) 76(3 Suppl):S94–100. doi: 10.1016/j.ijrobp.2009.06.092 20171524 PMC4388033

[9] Lee JH Kim DH Ki YK Nam JH Heo J Woo HY. Three-dimensional conformal radiotherapy for portal vein tumor thrombosis alone in advanced hepatocellular carcinoma. Radiat Oncol J (2014) 32(3):170–8. doi: 10.3857/roj.2014.32.3.170 PMC4194300 25324989

[10] Rim CH Seong J. Application of radiotherapy for hepatocellular carcinoma in current clinical practice guidelines. Radiat Oncol J (2016) 34(3):160–7. doi: 10.3857/roj.2016.01970 PMC5066447 27730805

[11] Bujold A Massey CA Kim JJ Brierley J Cho C Wong RK. Sequential phase I and II trials of stereotactic body radiotherapy for locally advanced hepatocellular carcinoma. J Clin Oncol (2013) 31(13):1631–9. doi: 10.1200/JCO.2012.44.1659 23547075

[12] Feng M Suresh K Schipper MJ Bazzi L Ben-Josef E Matuszak MM. Individualized adaptive stereotactic body radiotherapy for liver tumors in patients at high risk for liver damage: A phase 2 clinical trial. JAMA Oncol (2018) 4(1):40–7. doi: 10.1001/jamaoncol.2017.2303 PMC5766368 28796864

[13] Kim TH Koh YH Kim BH Kim MJ Lee JH Park B. Proton beam radiotherapy vs. radiofrequency ablation for recurrent hepatocellular carcinoma: A randomized phase III trial. J Hepatol (2021) 74(3):603–12. doi: 10.1016/j.jhep.2020.09.026 33031846

[14] Wahl DR Stenmark MH Tao Y Pollom EL Caoili EM Lawrence TS. Outcomes after stereotactic body radiotherapy or radiofrequency ablation for hepatocellular carcinoma. J Clin Oncol (2016) 34(5):452–9. doi: 10.1200/JCO.2015.61.4925 PMC4872011 26628466

[15] Yoo GS Yu JI Park HC. Proton therapy for hepatocellular carcinoma: Current knowledges and future perspectives. World J Gastroenterol (2018) 24(28):3090–100. doi: 10.3748/wjg.v24.i28.3090 PMC6064962 30065555

[16] Korean Liver Cancer A National Cancer Center GK. 2018 Korean Liver cancer association-national cancer center Korea practice guidelines for the management of hepatocellular carcinoma. Korean J Radiol (2019) 20(7):1042–113. doi: 10.3348/kjr.2019.0140 PMC6609431 31270974

[17] Shao YY Wang SY Lin SM Diagnosis G Systemic Therapy G. Management consensus guideline for hepatocellular carcinoma: 2020 update on surveillance, diagnosis, and systemic treatment by the Taiwan liver cancer association and the gastroenterological society of Taiwan. J Formos Med Assoc (2021) 120(4):1051–60. doi: 10.1016/j.jfma.2020.10.031 33199101

[18] Korean Liver Cancer Study G National Cancer Center K. [Practice guidelines for management of hepatocellular carcinoma 2009]. Korean J Hepatol (2009) 15(3):391–423. doi: 10.3350/kjhep.2009.15.3.391 19783891

[19] Korean Liver Cancer Study G National Cancer Center K. 2014 KLCSG-NCC Korea practice guideline for the management of hepatocellular carcinoma. Gut Liver (2015) 9(3):267–317. doi: 10.5009/gnl14460 25918260 PMC4413964

[20] Yu JI Park HC Yoo GS Choi C Choi MS Nam H. Clinical importance of the absolute count of neutrophils, lymphocytes, monocytes, and platelets in newly diagnosed hepatocellular carcinoma. Sci Rep (2021) 11(1):2614. doi: 10.1038/s41598-021-82177-5 33510378 PMC7844216

[21] Sinn DH Choi G-S Park HC Kim JM Kim H Song KD. Multidisciplinary approach is associated with improved survival of hepatocellular carcinoma patients. PloS One (2019) 14(1):e0210730. doi: 10.1371/journal.pone.0210730 30640924 PMC6331107

[22] Royston P Parmar MK. Restricted mean survival time: an alternative to the hazard ratio for the design and analysis of randomized trials with a time-to-event outcome. BMC Med Res Methodol (2013) 13:152. doi: 10.1186/1471-2288-13-152 24314264 PMC3922847

[23] Lawrence TS Robertson JM Anscher MS Jirtle RL Ensminger WD Fajardo LF. Hepatic toxicity resulting from cancer treatment. Int J Radiat Oncol Biol Phys (1995) 31(5):1237–48. doi: 10.1016/0360-3016(94)00418-K 7713785

[24] Jang WI Bae SH Kim MS Han CJ Park SC Kim SB. A phase 2 multicenter study of stereotactic body radiotherapy for hepatocellular carcinoma: Safety and efficacy. Cancer (2020) 126(2):363–72. doi: 10.1002/cncr.32502 31747476

[25] Kim JW Kim DY Han KH Seong J. Phase I/II trial of helical IMRT-based stereotactic body radiotherapy for hepatocellular carcinoma. Dig Liver Dis (2019) 51(3):445–51. doi: 10.1016/j.dld.2018.11.004 30503296

[26] Kimura T Takeda A Sanuki N Ariyoshi K Yamaguchi T Imagumbai T. Multicenter prospective study of stereotactic body radiotherapy for previously untreated solitary primary hepatocellular carcinoma: The STRSPH study. Hepatol Res (2021) 51(4):461–71. doi: 10.1111/hepr.13595 33217113

[27] Takeda A Sanuki N Tsurugai Y Iwabuchi S Matsunaga K Ebinuma H. Phase 2 study of stereotactic body radiotherapy and optional transarterial chemoembolization for solitary hepatocellular carcinoma not amenable to resection and radiofrequency ablation. Cancer (2016) 122(13):2041–9. doi: 10.1002/cncr.30008 27062278

[28] Fukumitsu N Sugahara S Nakayama H Fukuda K Mizumoto M Abei M. A prospective study of hypofractionated proton beam therapy for patients with hepatocellular carcinoma. Int J Radiat Oncol Biol Phys (2009) 74(3):831–6. doi: 10.1016/j.ijrobp.2008.10.073 19304408

[29] Kim TH Park JW Kim BH Oh ES Youn SH Moon SH. Phase II study of hypofractionated proton beam therapy for hepatocellular carcinoma. Front Oncol (2020) 10:542. doi: 10.3389/fonc.2020.00542 32411594 PMC7198869

[30] Su TS Liang P Liang J Lu HZ Jiang HY Cheng T. Long-term survival analysis of stereotactic ablative radiotherapy versus liver resection for small hepatocellular carcinoma. Int J Radiat Oncol Biol Phys (2017) 98(3):639–46. doi: 10.1016/j.ijrobp.2017.02.095 28581406

[31] Kim N Cheng J Jung I Liang J Shih YL Huang WY. Stereotactic body radiation therapy vs. radiofrequency ablation in Asian patients with hepatocellular carcinoma. J Hepatol (2020) 73(1):121–9. doi: 10.1016/j.jhep.2020.03.005 32165253

[32] Network NCC. Hepatobiliary cancers (version 4.2021). National Comprehensive Cancer Network (2021) Available: https://www.nccn.org/professionals/physician_gls/pdf/hepatobiliary.pdf.

[33] Apisarnthanarax S Barry A Cao M Czito B DeMatteo R Drinane M. External beam radiation therapy for primary liver cancers: An ASTRO clinical practice guideline. Pract Radiat Oncol (2022) 12(1):28–51. doi: 10.1016/j.prro.2021.09.004 34688956

[34] Tandon P Garcia-Tsao G. Prognostic indicators in hepatocellular carcinoma: a systematic review of 72 studies. Liver Int (2009) 29(4):502–10. doi: 10.1111/j.1478-3231.2008.01957.x PMC2711257 19141028

[35] Tangkijvanich P Mahachai V Suwangool P Poovorawan Y. Gender difference in clinicopathologic features and survival of patients with hepatocellular carcinoma. World J Gastroenterol (2004) 10(11):1547. doi: 10.3748/wjg.v10.i11.1547 15162522 PMC4572751

[36] Yoon SM Ryoo BY Lee SJ Kim JH Shin JH An JH. Efficacy and safety of transarterial chemoembolization plus external beam radiotherapy vs sorafenib in hepatocellular carcinoma with macroscopic vascular invasion: A randomized clinical trial. JAMA Oncol (2018) 4(5):661–9. doi: 10.1001/jamaoncol.2017.5847 PMC5885246 29543938

