# Supplementary figures and images for: Trends in radiotherapy administration in the management of hepatocellular carcinoma: Analysis of a Korean tertiary hospital registry of hepatocellular carcinoma patients diagnosed between 2005 and 2017
Source: Front Oncol. 2022 Jul 22;12:928119. doi: 10.3389/fonc.2022.928119 (PMC9355731; doi:10.3389/fonc.2022.928119)

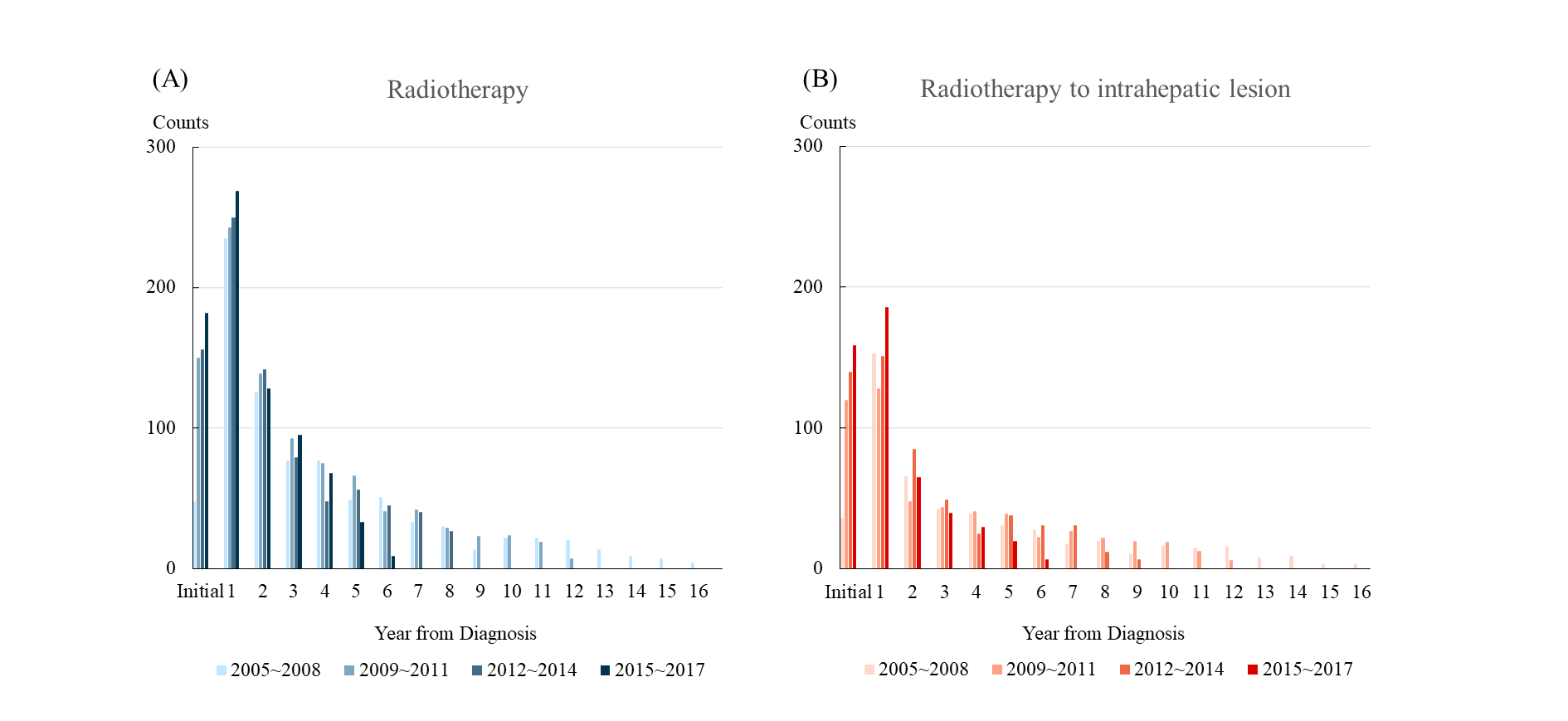

Supplement: Supplementary Figure 1 — Differential counts of radiotherapy utilization based on time from diagnosis. (A) radiotherapy to all lesions; (B) radiotherapy to intrahepatic lesions. [file Image_1.tif]

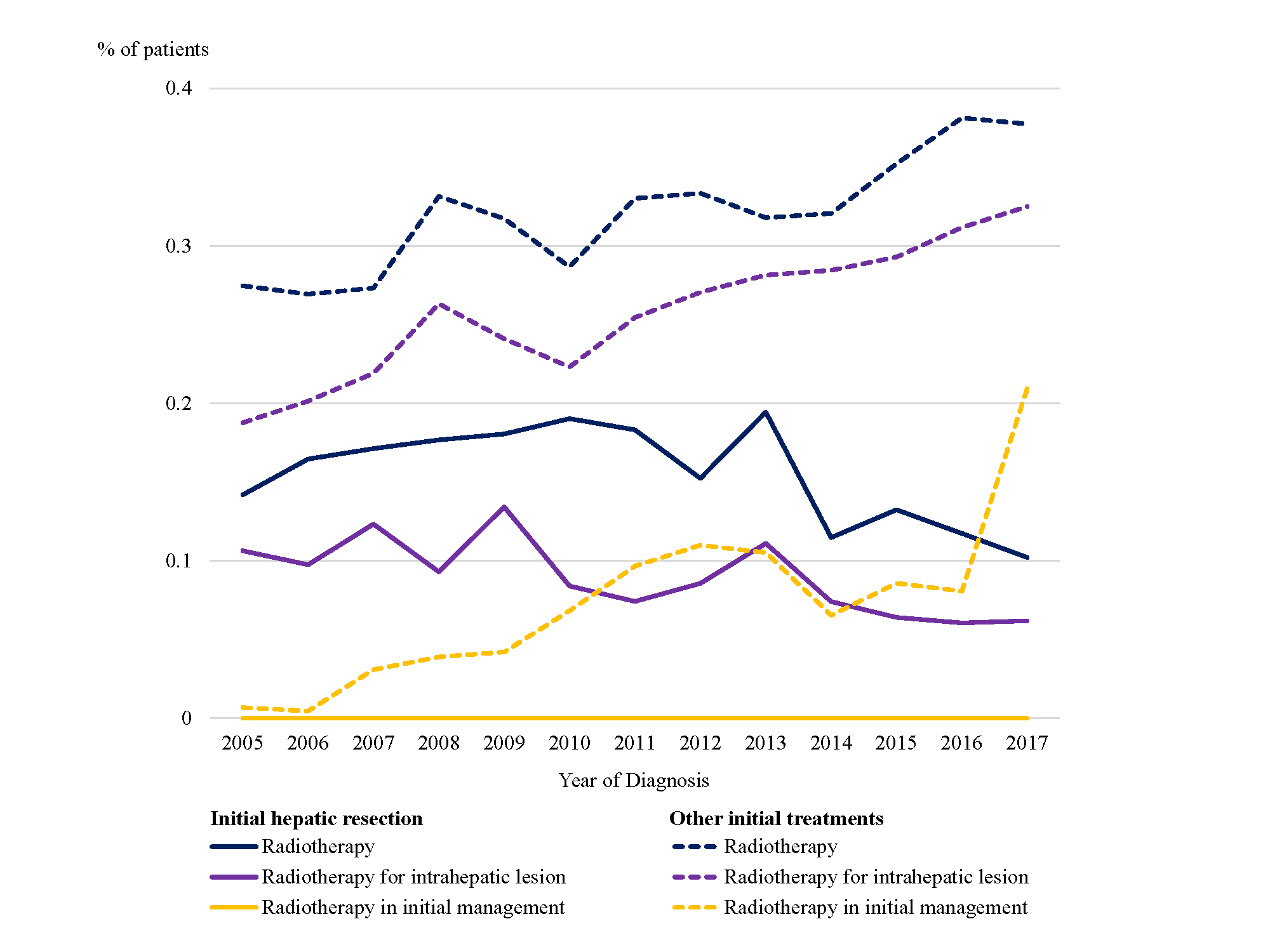

Supplement: Supplementary Figure 2 — Trends in radiotherapy utilization rate with patients grouped based on initial treatment: initial hepatic resection or other initial treatments. Trends of initial hepatic resection group is shown in solid line, and other initial treatment group is shown in dotted line. [file Image_2.tif]
