# Supplementary material for: Trends in radiotherapy administration in the management of hepatocellular carcinoma: Analysis of a Korean tertiary hospital registry of hepatocellular carcinoma patients diagnosed between 2005 and 2017
Source: Front Oncol. 2022 Jul 22;12:928119. doi: 10.3389/fonc.2022.928119 (PMC9355731; doi:10.3389/fonc.2022.928119)
Supplement: Supplementary file 4 [file DataSheet_1.docx]

Supplementary Table 1. Baseline clinical characteristics compared by radiotherapy utilization

| Characteristics | Total  N = 9,132 | No radiotherapy  n = 6,687 | Radiotherapy  n = 2,445 | P-value |
| --- | --- | --- | --- | --- |
| Sex |  |  |  | <0.001 |
| Male | 7,302 (80.0%) | 5,238 (78.3%) | 2,064 (84.4%) |  |
| Female | 1,830 (20.0%) | 1,449 (21.7%) | 381 (15.6%) |  |
| ECOG PS |  |  |  | <0.001 |
| 0 | 8,365 (91.6%) | 6,133 (91.7%) | 2,232 (91.3%) |  |
| 1 | 609 (6.7%) | 427 (6.4%) | 182 (7.4%) |  |
| 2 | 71 (0.8%) | 58 (0.9%) | 13 (0.5%) |  |
| 3 | 58 (0.6%) | 42 (0.6%) | 16 (0.7%) |  |
| 4 | 28 (0.3%) | 26 (0.4%) | 2 (0.1%) |  |
| Etiology |  |  |  | <0.001 |
| HBV | 6,704 (73.4%) | 4,843 (72.4%) | 1,861 (76.1%) |  |
| HBV+HCV | 113 (1.3%) | 80 (1.2%) | 33 (1.3%) |  |
| HCV | 868 (9.5%) | 661 (9.9%) | 207 (8.5%) |  |
| Alcohol | 566 (6.2%) | 425 (6.4%) | 141 (5.8%) |  |
| NBNC | 841 (9.2%) | 647 (9.7%) | 194 (7.9%) |  |
| Others | 26 (0.3%) | 20 (0.3%) | 6 (0.2%) |  |
| N/A | 14 (0.1%) | 11 (0.2%) | 3 (0.1%) |  |
| Diagnosis |  |  |  | 0.013 |
| Clinical diagnosis | 8,640 (94.6%) | 6,353 (95.0%) | 2,287 (93.5%) |  |
| Pathological diagnosis | 492 (5.4%) | 334 (5.0%) | 158 (6.5%) |  |
| ALBI grade |  |  |  | <0.001 |
| I | 5,363 (58.7%) | 3,864 (57.8%) | 1,499 (61.3%) |  |
| II | 3,398 (37.2%) | 2,491 (37.3%) | 907 (37.1%) |  |
| III | 371 (4.1%) | 327 (4.9%) | 44 (1.8%) |  |
| Child-Pugh classification |  |  |  | <0.001 |
| A | 7,848 (86.0%) | 5,636 (84.3%) | 2,212 (90.5%) |  |
| B | 1,127 (12.3%) | 904 (13.5%) | 223 (9.1%) |  |
| C | 157 (1.7%) | 142 (2.1%) | 15 (0.4%) |  |
| mUICC stage |  |  |  | <0.001 |
| I | 2,055 (22.5%) | 1,751 (26.2%) | 304 (12.4%) |  |
| II | 3,565 (39.0%) | 2,927 (43.8%) | 768 (31.4%) |  |
| III | 2,089 (22.9%) | 1,292 (19.3%) | 795 (32.5%) |  |
| IVA | 898 (9.8%) | 488 (7.3%) | 408 (16.7%) |  |
| IVB | 525 (5.8%) | 357 (5.3%) | 168 (6.9%) |  |
| BCLC stage |  |  |  | <0.001 |
| 0 | 1,749 (19.1%) | 1,485 (22.2%) | 264 (10.8%) |  |
| A | 3,805 (41.7%) | 2,959 (44.3%) | 846 (34.6%) |  |
| B | 993 (10.9%) | 653 (9.8%) | 340 (13.9%) |  |
| C | 2,360 (25.8%) | 1,398 (20.9%) | 962 (39.3%) |  |
| D | 225 (2.5%) | 192 (2.9%) | 33 (1.3%) |  |
| Initial Treatment |  |  |  | <0.001 |
| No treatment | 373 (4.1%) | 368 (5.5%) | 5 (0.2%) |  |
| Liver transplantation | 151 (1.7%) | 140 (2.1%) | 11 (0.4%) |  |
| Resection | 2,862 (31.3%) | 2,411 (36.1%) | 451 (18.4%) |  |
| Ablation | 1,664 (18.2%) | 1,371 (20.5%) | 293 (12.0%) |  |
| Combined local treatment | 150 (1.6%) | 124 (1.9%) | 26 (1.1%) |  |
| TACE | 3,002 (32.9%) | 1,928 (28.8%) | 1,074 (43.9%) |  |
| Combined treatment | 477 (5.2%) | 18 (0.3%) | 459 (18.8%) |  |
| Systemic treatment | 334 (3.7%) | 284 (4.2%) | 50 (2.0%) |  |
| Sole radiotherapy | 76 (0.8%) | 0 (0.0%) | 76 (3.0%) |  |
| Others | 34 (0.4%) | 31 (0.5%) | 3 (0.1%) |  |

ECOG PS, Eastern Cooperative Oncology Group performance status; HBV, hepatitis B virus; HCV, hepatitis C virus; NBNC, non-B non-C hepatitis; ALBI, albumin-bilirubin; mUICC, modified Union for International Cancer Control; BCLC, Barcelona Clinic Liver Cancer; TACE, transarterial chemoembolization

Supplementary Table 2. BCLC stage and the utilized radiotherapy technique.

| BCLC stage | 2D | 3D-CRT | IMRT | SBRT | PBT |
| --- | --- | --- | --- | --- | --- |
| 0 | 14 (5.5%) | 129 (7.5%) | 29 (10.9%) | 63 (20.2%) | 78 (22.5%) |
| A | 87 (34.3%) | 545 (31.8%) | 115 (43.1%) | 135 (43.3%) | 146 (42.2%) |
| B | 40 (15.7%) | 227 (13.2%) | 36 (13.5%) | 43 (13.8%) | 59 (17.1%) |
| C | 107 (42.1%) | 788 (46.0%) | 86 (32.2%) | 69 (22.1%) | 62 (17.9%) |
| D | 6 (2.4%) | 25 (1.5%) | 1 (0.4%) | 2 (0.6%) | 1 (0.3%) |

BCLC, Barcelona Clinic Liver Cancer; 3D-CRT, 3D-conformal radiotherapy; IMRT, intensity modulated radiotherapy; SBRT, streotactic body radiotherapy; PBT, proton beam therapy

Supplementary Table 3. Results of the Cochran–Armitage test for radiotherapy utilization.

| Year | 2005 | 2006 | 2007 | 2008 | 2009 | 2010 | 2011 | 2012 | 2013 | 2014 | 2015 | 2016 | 2017 | P value |  |  |  |  |  |  |  |  |  |  |  |  |  |
| --- | --- | --- | --- | --- | --- | --- | --- | --- | --- | --- | --- | --- | --- | --- | --- | --- | --- | --- | --- | --- | --- | --- | --- | --- | --- | --- | --- |
|  | N=578 | N=591 | N=534 | N=727 | N=714 | N=840 | N=823 | N=783 | N=746 | N=713 | N=572 | N=710 | N=800 |  |  |  |  |  |  |  |  |  |  |  |  |  |  |
| Radiotherapy  utilization | | | | | | | | | | | | | | 0.239 |  |  |  |  |  |  |  |  |  |  |  |  |  |
|  | 140  (24.2%) | 142 (24.0%) | 131 (24.5%) | 208 (28.6%) | 197 (27.6%) | 219 (26.1%) | 242 (29.4%) | 223 (28.5%) | 206 (27.6%) | 173 (24.3%) | 150 (26.2%) | 201 (28.3%) | 213 (26.6%) |  |  |  |  |  |  |  |  |  |  |  |  |  |  |
|  | 438  (75.8%) | 449 (76.0%) | 403 (75.5%) | 519 (71.4%) | 517 (72.4%) | 621 (73.9%) | 581 (70.6%) | 560 (71.5%) | 540 (72.4%) | 540 (75.7%) | 422 (73.8%) | 509 (71.7%) | 587 (73.4%) |  |  |  |  |  |  |  |  |  |  |  |  |  |  |
| Radiotherapy for  intrahepatic lesion | | | | | | | | | | | | | | 0.006 |  |  |  |  |  |  |  |  |  |  |  |  |  |
|  | 97 (16.8%) | 102 (17.3%) | 103 (19.3%) | 155 (21.3%) | 149 (20.9%) | 156 (18.6%) | 173 (21.0%) | 173 (22.1%) | 167 (22.4%) | 146 (20.5%) | 114 (19.9%) | 155 (21.8%) | 175 (21.9%) |  |  |  |  |  |  |  |  |  |  |  |  |  |  |
|  | 481  (83.2%) | 489 (82.7%) | 431 (80.7%) | 572 (78.7%) | 565 (79.1%) | 684 (81.4%) | 650 (79.0%) | 610 (77.9%) | 579 (77.6%) | 567 (79.5%) | 458 (80.1%) | 555 (78.2%) | 625 (78.1%) |  |  |  |  |  |  |  |  |  |  |  |  |  |  |
| Radiotherapy as  initial management | | | | | | | | | | | | | | <0.001 |  |  |  |  |  |  |  |  |  |  |  |  | < 0.001 |
|  | 3  (0.5%) | 2 (0.3%) | 12 (2.2%) | 20 (2.8%) | 21 (2.9%) | 42 (5.0%) | 60 (7.3%) | 63 (8.0%) | 52 (7.0%) | 29 (4.1%) | 29 (5.1%) | 36 (5.1%) | 100 (12.5%) |  |  |  |  |  |  |  |  |  |  |  |  |  |  |
|  | 575  (99.5%) | 589 (99.7%) | 522 (97.8%) | 707 (97.2%) | 693 (97.1%) | 798 (95.0%) | 763 (92.7%) | 720 (92.0%) | 694 (93.0%) | 684 (95.9%) | 543 (94.9%) | 674 (94.9%) | 700 (87.5%) |  |  |  |  |  |  |  |  |  |  |  |  |  |  |

| Author | Patients | CP – A  (%) | Radiotherapy | Dose/  Fractionation | 1yr LC  (%) | 2yr LC  (%) | 3yr LC  (%) | 1yr OS  (%) | 2yr OS  (%) | 3yr OS  (%) | Toxicity  (≥ G3, N) |
| --- | --- | --- | --- | --- | --- | --- | --- | --- | --- | --- | --- |
| Kimura et al.[25] | 36 | 91 | SBRT | 40 Gy/5 Fx |  | 90 | 90 |  | 84 | 78 | 4 (11.1%) |
| Jang et al.[23] | 65 | 98.5 | SBRT | 60-45 Gy/3 Fx |  | 97 | 95 |  | 84 | 76 | 2 (3.1%) |
| Kim et al.[24] | 32 | 87.5 | SBRT | 36-60 Gy/4 Fx | 90.6 | 84.2 |  | 93.8 | 81.3 |  | 0 (0.0%) |
| Takeda et al.[26] | 90 | 91 | SBRT | 35-40 Gy/5 Fx |  |  | 96.3 |  |  | 66.7 | 10 (11.1%) |
| Fukumitsu et al.[27] | 51 | 80.4 | PBT | 66 GyE/10 Fx |  |  | 94.5 |  |  | 49.2 | 0 (0.0%) |
| Kim et al.[28] | 45 | 100 | PBT | 70 GyE/10 Fx |  |  | 95.2 |  |  | 86.4 | 0 (0.0%) |

Supplementary Table 4. Summary of prospective clinical trials of radiotherapy in management of intrahepatic lesions of hepatocellular carcinoma.

CP-A, Child-Pugh classification A; LC, local control; OS, overall survival; SBRT, stereotactic body radiotherapy; PBT, proton beam therapy; Gy, grays; Fx, fractions; GyE, gray equivalent
